# Impact of tapering and proactive recovery on young elite rugby union players’ repeated high intensity effort ability

**DOI:** 10.5114/biolsport.2022.109453

**Published:** 2021-09-30

**Authors:** Adrien Vachon, Nicolas Berryman, Iñigo Mujika, Jean-Baptiste Paquet, Fabien Sauvet, Laurent Bosquet

**Affiliations:** 1 Lab MOVE (EA6314), Faculty of sport sciences, University of Poitiers, 8 allée Jean Monnet (bât C6), TSA 31113, 86073 Poitiers cedex 9, France; 2 Stade Rochelais Rugby, 27 Avenue du Maréchal Juin, 17000 La Rochelle, France; 3 Département des sciences de l’activité physique, 141 Avenue du Président Kennedy, Université du Québec à Montréal, Montréal (Qc), Canada H2X 1Y4; 4 Institut national du sport du Québec, 4141 Pierre de Coubertin, Montréal (Qc), Canada H1V 3N7; 5 Department of Sports Studies, Bishop’s University, 2600 College, Sherbrooke (Qc), Canada J1M 1Z7; 6 Department of Physiology, Faculty of Medicine and Nursing, University of the Basque Country, Leioa, Basque Country; 7 Exercise Science Laboratory, School of Kinesiology, Faculty of Medicine, Universidad Finis Terrae, Santiago, Chile; 8 Institut de recherche biomédicale des armées (IRBA), Brétigny sur orge, France EA7330 VIFASOM, Université de Paris, Hotel Dieu, Paris, France; 9 Department of kinesiology, University of Montreal, CP6128 succ. centre ville. Montreal (Qc), Canada H3C 3J7

**Keywords:** Supercompensation, Repeated efforts, Sleep, Fatigue, Cold-water immersion, Foam rolling, Training load

## Abstract

To assess the effects of a taper combined with proactive recovery on the repeated high intensity effort (RHIE) of elite rugby union players, and the possible interaction of pre-taper fatigue and sleep. Eighteen players performed a 3-week intensive training block followed by a 7-day exponential taper combined with a multicomponent recovery strategy. Following the intervention, players were divided into 3 groups (Normal Training: NT, Acute Fatigue: AF or Functional Overreaching: F-OR) based on their readiness to perform prior to the taper. Total sprint time [TST], percentage decrement [%D] and the number of sprints ≥90% of the best [N90] were analyzed to assess performance during a RHIE test. Subjective sleep quality was assessed through the Pittsburg Sleep Quality Index (PSQI) and the Epworth Sleepiness Scale (ESS). No improvement in TST was reported in either NT or F-OR after the taper, whereas AF tended to improve (-1.58 ± 1.95%; p > 0.05; g = -0.20). F-OR players reported baseline PSQI and ESS indicative of sleep disturbance (6.2 ± 2.2 and 10.6 ± 5.4, respectively). AF displayed a small impairment in PSQI during intensive training (11.5 ± 80.6%; p > 0.05; g = 0.20), which was reversed following the taper (-34.6 ± 62.1%; p > 0.05; g = -0.73). Pre-taper fatigue precluded the expected performance benefits of the combined taper and recovery intervention, likely associated with a lack of strictly controlled intensive training block. Poor sleep quality before the intensive training period appeared to predispose the players to developing functional overreaching.

## INTRODUCTION

The potential benefits of a taper on performance are well established, both in endurance and team sports [1, 2]. Studies in individual sports suggest that performance improvement following a taper is bigger when it is preceded by a period of overload training, provided that the level of accumulated fatigue is moderate (i.e. Acute Fatigue – AF). Indeed, a taper did not enhance performance when athletes developed a state of functional overreaching (F-OR) [3, 4]. These observations suggest the existence of an inverted U relationship between pre-taper fatigue and taper-induced improvements. A recent study on elite rugby union players [5] found that players showing a moderate level of fatigue after a 3-week intensive training block had greater improvement in repeated high intensity effort ability (RHIE) than players reporting a high level of fatigue, as determined by psychometric questionnaires, neuromuscular performances, and submaximal heart rate tests. The authors hypothesized that such performance difference could be explained by an insufficient decrease in training load during the 7-day taper to recover from the higher level of fatigue, and highlighted that congested schedules predispose the players to developing more severe states of fatigue [5]. If training volume during the taper is decreased insufficiently or the taper is not long enough to allow full recovery and performance supercompensation, the taper could be complemented with proactive recovery strategies. Indeed, LeMeur et al. [6] reported larger taper effects on an incremental running test in triathletes who combined a 1-week taper with whole body cryotherapy, highlighting the importance of optimizing recovery when it is only possible to implement a short-duration taper.

Sleep is essential for recovery because it stimulates anti-inflammatory and antioxidant responses, the production of anabolic hormones facilitating cellular and tissue repair, and promotes the elimination of waste products [7]. However, athletes exhibit more sleep disorders than the common population, especially during phases of high training load and competition [8]: 50–78% of elite athletes experienced sleep disturbance and 22–26% a highly disturbed sleep. Few days of partial sleep restriction could lead to an increased risk of injury and illness [9], increase perceived difficulty and an alteration of physiological performance [10]. To counterbalance the effect of sleep disruption or improve sleep quality, few strategies are available to players: water immersion [11], sleep hygiene education [12] or innovative mattresses [13] have been reported to be effective for rugby union players.

In this context, it could be appropriate to implement a recovery plan targeting sleep quality, wellbeing, inflammation and muscle damage when accelerated recovery is needed (i.e. short duration taper) [14]. In the demanding context of elite rugby union, the inclusion of a multi component recovery strategy could facilitate taper-induced adaptations and performance improvements, especially in players reporting more severe states of accumulated fatigue and impaired sleep.

This study aimed to assess the effects of a taper combined with proactive recovery methods on the RHIE ability of young elite rugby union players following an intensive training block. We hypothesized that such a strategy would allow F-OR players to benefit from a taper as much as AF players. Considering the physiological and psychological role of sleep on recovery [15], a secondary purpose was to assess the quality of sleep during intensive training and tapering, in order to verify the role of sleep in the supercompensation process.

## MATERIALS AND METHODS

### Participants

Participants were 24 members of the U21 team from the same Top 14 (1st division of French professional rugby union) club. They played at the top national level and regularly joined the professional team. Six participants withdrew from the study due to moderate injuries (unavailability > 3 days). The final sample size was 18 players (age 19 ± 1 years; height 181.6 ± 8.1 cm; body mass 89.8 ± 13.9 kg). All procedures were part of the teams’ service provision, which conforms to the Code of Ethics of the World Medical Association (Declaration n°IRB00012476-2020-10-10-66). Athletes provided informed consent to participate in monitoring procedures associated with team duties, with the understanding that data may be used for research purposes.

### Experimental design

**FIG. 1:**
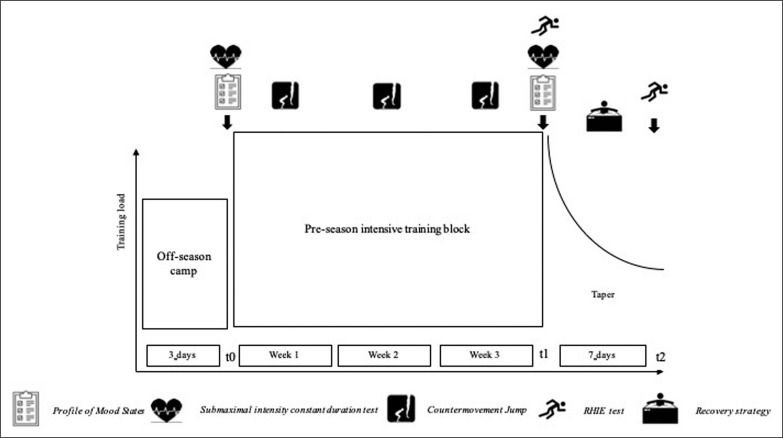
Schematic representation of the study design.

All players followed the same 3-week pre-season intensive training block followed by a 7-day exponential taper (Figure 1). Intensive training was characterized by a high training load, and a stable percentage distribution of training contents (i.e. neuromuscular, cardiovascular, technical/tactical training). Players underwent 15 ± 4 training sessions per week over 5 training days, with a weekly perceived effort of 6.5 ± 0.2 on the CR-10 scale (Table 1).[16] The 7-day taper consisted of a 58 ± 5% exponential decrease of training volume with a 30% decrease in training frequency. To enhance players’ recovery during the taper, a proactive recovery plan including a sleep hygiene intervention, the use of innovative high-heat capacity mattresses, foam rolling and cold-water immersion was implemented. The sleep hygiene intervention was conducted during intensive training. During the taper, training days ended with a 20-min foam rolling session, immediately followed by 10-min of cold-water immersion. Participants were also required to sleep on an innovative high-heat capacity mattress (≈6 nights). Performance changes were assessed with a rugby-specific RHIE test performed after intensive training (t1) and after the taper (t2). Players were *a posteriori* classified in fatigue groups based on their readiness to perform prior to the taper. Readiness to perform was assessed with a psychometric questionnaire (Profile of Mood States–POMS), a submaximal constant duration cycling test to measure heart rate (HR), and a neuromuscular test (Countermovement jump-CMJ). The questionnaire and the constant duration test were completed at baseline (t0) and t1, while the neuromuscular test was performed thrice weekly during intensive training. Moreover, to verify the interaction of sleep with recovery and the taper supercompensation process, sleep quality was assessed through the Epworth Sleepiness Scale (ESS) and Pittsburgh Sleep Quality Index (PSQI) at t0, t1 and t2.

**TABLE 1:** Frequency, volume and intensity parameters of training bloc and taper period.

			Training block		Taper
**Duration (weeks)**		1	1	1	1
**Training load (a.u.)**		3630 ± 154	3416 ± 252^#^	3846 ± 340^!^	1466 ± 174^a^
**Perceived effort (CR-10)**		7.4 ± 0.5	6.3 ± 0.5^#^	6.1 ± 0.7^#!^	5.3 ± 0.5^a^
**Training distribution (%) (N/C/T)**		61/30/9	58/16/26	44/10/46	48/11/41^a^
**Total distance (m)**		16840 ± 1501	20393 ± 2466^#^	23109 ± 3196^#!^	11731 ± 556^a^
**Distance > 20 km**·**h^-1^ (m)**		1334 ± 471	1208 ± 874	1037 ± 586	1045 ± 465^a^
**Distance > 20 km**·**h^-1^ (%)**		8 ± 2	5 ± 4^#^	4 ± 2^#^	9 ± 4^a^

**Technical and tactical training**	Number of sessions (n) Duration (min)	1 45	5 140	7 310	4^a^ 125^a^

**Neuromuscular training**	Number of sessions (n) Duration (min) Number of reps (n)	6 300 530	7 315 544	7 292 542	4^a^ 150^a^ 278^a^

**Cardiovascular training**	Number of sessions (n) Duration (min)	4 147	3 85	3 65	2^a^ 35^a^

Note: a.u.: arbitrary units; N = Neuromuscular training; C = Cardiovascular training; T = technical and tactical training; HI running = High intensity running;

# Different from week 1 with p < 0.05;

! Different from week 2 with p < 0.05;

a Different from the 3-week training block average with p < 0.05

### Tests and measures

#### Training load

Training load was assessed using the session rating of perceived exertion (sRPE)[17]. Approximately 30 min after each training session, participants recorded the perceived effort on the CR-10 scale. Daily training load was obtained by multiplying the average perceived effort by training duration (minutes). Weekly training load was obtained from the sum of daily training loads. All participants had experience with the CR-10 for at least 6 months.

Rugby training volume and intensity were monitored using GPS units (Gpexe pro^2^, Exelio Srl, Udine, Italy) placed in a small pocket of a wearable shirt, between the two scapulae. Total distance (meters) and distance covered at velocities > 20 km·h^-1^ were retained for further assessments [18]. The distance covered at velocities > 20km·h^-1^ was also expressed as a percentage of total distance (%HI). During neuromuscular training sessions, volume was monitored through the number of repetitions performed.

### Recovery plan

#### Foam Rolling

Immediately after the end of the last training session of the day (≈4:30PM) participants underwent a 20-min self-myofascial release session with a foam roller (Performance Roller, PhysioSupplies, Groningen, Netherlands) on the quadriceps, hamstrings, iliotibial band, calves, adductors and latissimus-dorsi, according to Healy et al. [19]. Participants were told to place as much of their body mass as possible onto the foam roller and to roll the foam cylinder from the top of the selected area to the bottom. Cadence was not standardized but two bouts of 45 s were performed on each muscle group.

#### Cold Water Immersion

Immediately after the foam rolling session and approximately 30 min after the end of the last training session of the day (≈5:00 PM), participants immersed their lower limbs in an ice bath at 12.0 ± 1.0°C, to the level of the iliac crest for 10 min according to Machado et al. [20].

#### Sleep hygiene intervention

The sleep hygiene intervention was conducted by an expert in sleep medicine. The intervention was composed of two sessions lasting ≈2 h each: ≈60 min of theoretical intervention followed by a 30-min nap with 5 min of guided relaxation techniques [21]. The first intervention emphasized the impact of sleep deprivation on athletes’ physical and psychological performance, as well as factors that are detrimental for sleep quality; the second session described strategies to enhance sleep quality (e.g. the implementation of a healthy and regular sleep routine, the use of napping, the use of caffeine.

#### Innovative mattress

During the 6 nights of the 7-day taper, participants added to their usual mattress a 6-cm polyurethane high-heat capacity layer with a density of 1.006 kg/m^3^, which allows the slow removal of body heat via conductive heat transfer [22].

### Performance

#### Repeated High Intensity Effort (RHIE) test

The procedure and reliability of the RHIE test (Figure 1) has been reported previously [23]. Total sprint time (TST) was considered the final test result, with a meaningful change threshold of 3.59%, and percentage decrement (%D) an indication of an athlete’s ability to delay fatigue. The number of sprints equal or higher than 90% of the best sprint (N90) was also recorded. Results were analyzed if the coefficient of variation (CV) of an athlete’s tackle performance was less than 45.9% and 34.2% for total g-force and average g-force, respectively. This verification ensures that a player produced a steady effort on the tackle task [23].

### Readiness to perform

#### Profile of Mood States questionnaire

The POMS [24] is a 65-item questionnaire that provides measures of vigor, depression, fatigue, anger, anxiety and confusion. The mood state index was obtained by adding the 5 negative factors together and subtracting the positive factor of vigor. The energy index (EI) represents the difference between the scores of vigor and fatigue [25]. Considering their sensitivity to overload- and taper-induced changes in performance [26] vigor, fatigue and EI were retained for subsequent analyses.

#### Submaximal intensity constant duration test

This test consisted of a 5-min submaximal intensity exercise on a cycle ergometer (Group Cycle Connect, Technogym, Cesena, Italy) immediately followed by 5 min of passive rest (5’-5’ Heart Rate Recovery test) during which players were asked to remain seated and HR was continuously monitored (Firstbeat SPORT, Jyväskylä, Finland) beat by beat during exercise and recovery. Proposed by Buchheit et al. [27] as a running test, it was adapted as a cycle ergometer test to facilitate its implementation within the athletes’ training environment. Players were familiar with the cycle ergometer as cycling sessions were part of the typical warm up before strength sessions. Exercise intensity was set at 200W for all players. Heart rate series were edited and visually inspected so that ectopic beats could be replaced by interpolated data from adjacent normal-to-normal intervals. The time constant t, which represents the time needed to reach 63% of the gain, and HR at the end of exercise were used for analysis. Submaximal HR was determined as the average HR value of the last minute of exercise.

#### Countermovement jump test

A countermovement jump was performed using a commercially available system (Optojump, Microgate Corporation, Bolzano-Bozen, Italy). All jumps were initiated from a standing still position, followed by a 90° knee flexion immediately followed by the jump phase. Players kept their hands on their waist during the entire jump. Each player performed three maximal CMJ interspersed by 1 min of rest and the average height of the 3 jumps was retained for analyses [28].

### Sleep quality

#### Pittsburg Sleep Quality Index

The Pittsburg Sleep Quality Index (PSQI)[29] is a 24-item scale which assesses the sleep disturbances through 7 items: subjective sleep quality, sleep latency, sleep duration, habitual sleep efficiency, sleep disturbances, use of sleep medication and daytime dysfunction. Items scores are added to obtain a global score. PSQI scores > 5 are considered sleep disturbance.

#### Epworth Sleepiness Scale

The Epworth Sleepiness Scale (ESS)[30] is a self-administered questionnaire consisting in 8 items about a subject’s likelihood of dozing or falling asleep in common life situations. ESS values > 10 (on a maximum of 24) are indicative of significant sleepiness; a score between 12 and 14 important sleepiness and scores > 14 severe sleepiness.

### Data analysis

#### Fatigue Cluster

To describe the magnitude of changes in psychological, cardiovascular and neuromuscular variables between t0 and t1, measures were expressed as a factor of the smallest worthwhile change, according to the procedure described by Vachon et al. [5]. Based on the magnitude of difference between t0 and t1, three researchers blinded to players’ characteristics independently assessed the fatigue level of participants as Normal Training (NT), Acute Fatigue (AF) or Functional Overreaching (F-OR). Selection criteria for these 3 scenarios are presented in Table 2. Changes in vigor, fatigue and EI were used to assess changes in mood states. Furthermore, an EI ≤ 0 was considered as a warning test value [25]. Agreement between all researchers was required to confirm the fatigue state of a player. The final categorization was validated by the senior strength and conditioning coach during the pre-season period. Any disagreement was discussed and resolved in a consensus meeting.

**TABLE 2:** Selection criteria of Normal training, Acute fatigue and Functional Overreaching group.

Number of scenarios	Psychological test	Neuromuscular test Cardiovascular test	Group
**1**	1 large or very large negative change	1 Moderate negative change OR Multiple small negative changes	F-OR

**2**	1 Moderate negative change OR EI warning test value	1 Small negative change	AF

**3**	1 Moderate negative change OR Multiple small negative changes	1 large or very large negative change	F-OR

**4**	Multiple trivial or positive changes		NT

Note: NT = Normal training; AF = Acute fatigue; F-OR = Functional Overreaching; EI warning test: energy index ≤ 0.

### Statistical analysis

Data are presented as means ± SD. Changes in psychological, cardiovascular and neuromuscular measures between t0 and t1 were expressed as a factor of the SWC. All data were log transformed to reduce bias arising from non-uniformity error. Data normality was evaluated by the Shapiro-Wilk test. Student *t* tests for dependent samples or Wilcoxon tests when normality of distribution was not satisfied were used to test the null hypothesis that measures were similar between t0 and t1. A 2-way factorial analysis of variance (group x time) with repeated measures on the time factor was performed to test the null hypothesis that measures were similar between groups and at each time point. Multiple comparisons were made with Tuckey HSD post-hoc test. Student *t* test for independent samples was performed to test the null hypothesis that difference between t1 and t2 (i.e. D t1/t2) was similar between groups. The magnitude of the difference between independent samples was assessed by Cohen’s d and by Hedge’s g for dependent samples. Cohen’s scale was used for interpretation of both measures where changes were considered small (0.20≤|Effect Size-ES| < 0.50), moderate (0.50≤|ES| < 0.80), or large (|ES|≥0.80) [31]. Statistical tests were conducted using Statistica (Version 10; StatSoft, Tulsa, USA).

## RESULTS

### Readiness to perform

Individual cardiovascular, neuromuscular and psychological adaptations to intensive training (t0–t1) are reported in Table 3. Six players (# 7, 9, 11, 13, 14, 15) met the criteria for Scenario 1, 8 players (# 1, 4, 5, 8, 10, 11, 13, 17) met the criteria for Scenario 2 whereas 7 players were associated with Scenario 4 (# 2, 3, 6, 12, 16, 17, 18). None of the players were associated to Scenario 3. Three players met the criteria for several scenarios and the senior strength and conditioning coach decision was used to validate the final categorization, leading to the inclusion of 6 players in the NT group, 7 players in the AF group and 5 in the F-OR group.

**TABLE 3:** Illustration of players pre- to post-training block changes and group decision making process.

Player	Psychological test				Cardiovascular test		Neuromuscular test					Decision Making	
	Fatigue	Vigor	Energy Changes	Index Raw	τ	HRex	Day 1	Day 2	Day 4	Day 6	Scenario	S&C coach assessment	Group
**1**	↑↑	-	↓↓	19	↑↑	↑	↓	↓↓		↑	2		AF
**2**	↓↓↓	-	↑↑↑	0				-	↑	↑	4		NT
**3**	↓↓	↓	↑	18	↑↑↑↑	↑↑↑	↑	↓		-	4		NT

**4**	↑↑	↓	↓↓	14			↑	↓	-	-	2		AF
**5**	↑↑	-	↓↓	6	↓↓↓	↑↑↑		↓	↓	↑	2		AF
**6**	↓↓↓	↑	↑↑↑	22	↓	-	↓	↓	↓	↑	4		NT

**7**	↑↑↑	↓	↓↓↓↓	9	-	↓↓	↓	↓↓	↓	↓	1		F-OR
**8**	↑↑	-	↓↓	9	↓↓	↓↓↓		↓		↑	2		AF
**9**	-	↓↓↓	↓↓↓	10	↓↓↓↓	↑	↓↓↓	-	-	↑↑↑	1		F-OR

**10**	↑↑↑↑	↑↑↑	↓↓	9			-	↓↓↓	-	↑	2		AF
**11**	↓↓↓	↑↑↑	↑↑↑↑	0		↓↓↓					2/1	F-OR	F-OR
**12**	-	↑	↑	17	-	↓↓↓	↓↓	↓	↑	↑	4		NT

**13**	1	↓↓↓	↓↓↓	7	↓↓↓	↓↓↓	-	↓	↓	↓	1 / 2	AF	AF
**14**	↑↑	↓↓↓↓	↓↓↓↓	-5	↓	↓↓↓↓	-	-	-	-	1		F-OR
**15**	-	↓↓↓	↓↓↓	12	↑	-	-	↓↓	-	-	1		F-OR

**16**	↑↑	↑↑	↑	10	↑↑↑	↑	-	↓	-	↑	4		NT
**17**	↑↑	↓	↓↓↓	17		↓↓↓	↑↑			↑	2/4	AF	AF
**18**	↓↓↓↓-	↓	↑↑↑↑	5			-	-	-	↑	4		NT

Note: - Trivial; ↑ Small increase; ↑↑ Moderate increase; ↑↑↑ Large increase; ↑↑↑↑ Very large increase; ↓ Small decrease; ↓↓ Moderate decrease; ↓↓↓ Large decrease; ↓↓↓↓ Very large decrease; NT = Normal training group; AF = Acute fatigue group; F-OR = Functional Overreaching; ȶ = time constant; HRex = Heart rate exercise.

### Taper effect on Repeated High Intensity Effort (RHIE) ability

Taper effects on RHIE ability are summarized in Table 4. There was no difference between groups at t1 in any RHIE indices. The overall group (n = 18) showed no improvement in TST. No improvement was reported in either NT or F-OR after the taper, whereas a tendency toward improvement was observed in AF. Concerning %D, the overall group show a small non-significant improvement after the taper, also observed in F-OR and AF, whereas NT decreased slightly. Regarding N90, no taper-induced improvement was found in the overall group, nor any interaction between groups. All players met the validation criterion of tackle indices.

**TABLE 4:** Taper effect on RHIE ability of elite young rugby union players.

	Group	t1	t2	t1/t2	
				Changes	ES (Hedges’ g)
TST (s)	Overall	40.45 ± 2.14	40.40 ± 2.36	-0.11 ± 2.18%	*-0.02 Trivial*
	NT	40.05 ± 2.16	40.34 ± 3.36	0.74 ± 1.28%	*0.11 Trivial*
	AF	40.74 ± 2.49	40.10 ± 2.75	-1.58 ± 1.95%	*-0.20 Small*
	F-OR	40.51 ± 2.00	40.89 ± 2.41	0.92 ± 2.50%	*0.13 Trivial*

%D (%)	Overall	7.39 ± 2.47	6.78 ± 2.69	-0.61 ± 2.55%	*-0.23 Small*
	NT	6.25 ± 2.33	7.06 ± 3.03	0.81 ± 2.52%	*0.25 Small*
	AF	8.05 ± 2.65	6.50 ± 2.93	-1.55 ± 2.11%	*-0.48 Small*
	F-OR	7.82 ± 2.39	6.82 ± 2.47	-1.00 ± 2.87%	*-0.33 Small*

N90 (n)	Overall	9.9 ± 2.7	10.3 ± 2.8	8.53 ± 32.3%	*0.13 Trivial*
	NT	10.2 ± 2.6	10.2 ± 3.1	2.90 ± 37.5%	*0.00 Trivial*
	AF	9.7 ± 3.3	10.3 ± 3.2	10.87 ± 25.6%	*0.15 Trivial*
	F-OR	9.8 ± 2.5	10.4 ± 2.6	12.00 ± 40.1%	*0.19 Trivial*

Note: TST = Total sprint time; %D = Percentage decrement; N90 = Number of sprint equal or superior to 90% of best sprint; NT = Normal training; AF = Acute fatigue; F-OR = Functional Overreaching; t0 = Start of the training bloc; t1 = Start of the taper; t2 = End of the taper.

### Intensive training and taper effects on sleep quality

Taper effects on subjective sleep quality are presented in Table 5. Overall, a tendency was found toward a small improvement in PSQI between t0 and t1, and t1 and t2. When assessing interaction between groups, no changes between time points were found for NT, whereas a small impairment between t0 and t1 was observed in AF, which was reversed during the taper. F-OR reported the highest PSQI values at t0 exceeding the minimal value (i.e. 5) considered as sleep disturbance. Following the overload period (i.e. t1), F-OR showed a large improvement in sleep quality, which was maintained during the taper.

**TABLE 5:** Taper effect on subjective sleep quality of elite young rugby union players.

	Group	t0	t1	t2	t0/t1	t1/t2
					Changes ES (Hedges’g)	Changes ES (Hedges’g)
**PSQI**	Overall	4.4 ± 2.0	3.8 ± 1.6	3.1 ± 1.4	-13.9 ± 55.3% *-0.32 Small*	-17.6 ± 56.0 % *-0.42 Small*
	NT	3.7 ± 1.9	3.3 ± 1.5	3.0 ± 0.9	-9.1 ± 22.3% *-0.15 Trivial*	-10.0 ± 24.5 % *-0.16 Trivial*
	AF	3.7 ± 1.4	4.1 ± 2.3	2.7 ± 1.0	11.5 ± 80.6% *0.20 Small*	-34.6 ± 62.1 % *-0.73 Moderate*
	F-OR	6.2 ± 2.2	3.8 ± 0.4	3.8 ± 2.2	-38.7 ± 35.3% *-1.21 Large*	0.0 ± 67.1% *0.00 Trivial*

**ESS**	Overall	9.6 ± 4.2	9.9 ± 4.8	8.8 ± 4.3	2.9 ± 24.4% *0.06 Trivial*	-11.2 ± 33.8 % *-0.23 Small*
	NT	8.8 ± 4.8	8.8 ± 5.5	8.7 ± 5.4	0.0 ± 10.1% *0.00 Trivial*	-1.9 ± 23.1% *-0.03 Trivial*
	AF	9.0 ± 3.4	10.6 ± 5.4	7.9 ± 4.3	17.5 ± 30.0% *0.22 Small*	-25.7 ± 28.2 *-0.46 Small*
	F-OR	11.4 ± 4.9	10.2 ± 3.9	10.2 ± 3.2	-10.5 ± 20.9% *-0.20 Small*	0.0 ± 45.5% *0.00 Trivial*

Note: PSQI = Pittsburg Sleep Quality Index; ESS = Epworth Sleepiness Scale; NT = Normal training; AF = Acute fatigue; F-OR = Functional Overreaching; t0 = Start of the training bloc; t1 = Start of the taper; t2 = End of the taper.

Concerning ESS, the overall group only reported a tendency toward a small improvement between t1 and t2. NT did not report any changes during the follow-up period, whereas a small impairment between t0 and t1 was observed in AF, with a value reaching the threshold (i.e. 10) to consider a daytime sleepiness. This score was reversed after the taper. F-OR reported the highest ESS values at t0 exceeding the value (i.e. 10) of daytime sleepiness. A tendency toward a small improvement was reported between t0 and t1, but values were still above daytime sleepiness at t1 and t2.

## DISCUSSION

This study aimed to assess the combined effect of a taper with a concomitant implementation of recovery methods on the RHIE ability of young elite rugby union players following an intensive training block. We hypothesized that the intervention would allow players diagnosed with F-OR to benefit from the same magnitude of improvement than players who exhibited an optimal accumulated fatigue (AF). Our main results were: 1) In the absence of a strict control of the training load periodization (i.e. training distribution and training intensity) during intensive training, the accumulated fatigue before the taper was not specific enough to obtain a benefit from the intervention; 2) nevertheless, magnitude-based and individual analyses revealed that sleep quality can be used to identify players at a greater risk of functional overreaching, and seems to play an important role in the players’ adaptation process.

### Taper effect on RHIE ability

Changes induced by the present intervention were too small to reach statistical significance (p < 0.05), but interesting tendencies still emerged. Although the overall group did not improve TST and N90 performance after the taper, a tendency was found for small benefits in %D. When comparing the magnitude of difference between groups, AF benefited from a small improvement in TST (-1.58 ± 1.95%, g = -0.20) and %D (-1.55 ± 2.11%, g = -0.48), while it remained unchanged in in NT and F-OR.

This observation is in accordance with previous studies in triathlon [3], distance running [4] and rugby union [5], which suggested that the highest magnitude of improvement following a taper was reached in the presence of moderate levels of accumulated fatigue. Nevertheless, the magnitude of improvement reported in AF (-1.58 ± 1.95%) was smaller than that previously reported by Vachon et al. [5] who found a 3.4 ± 3.9% improvement in TST following a 7-day taper without additional recovery strategies. This discrepancy between both protocols is surprising, since LeMeur et al. [6] reported a higher magnitude of improvement in peak velocity during an incremental test when whole body cryotherapy was added to the taper. These authors reported that the combination of a taper and recovery methods was particularly effective (4.2 ± 4.5% improvement) in the participants with the highest level of cumulative fatigue (i.e. functional overreaching).

The results of LeMeur et al. [6] contrast with the results of the present study, although our hypotheses were similar: an additive effect of recovery methods on the inflammation process, the sleep quality and the general well-being of players [14]. As discussed above, the main reason of such discrepancies likely relies on the lack of control of the intensive training, mainly due to the Covid-19 sanitary situation. Similar observations were made by Maestu et al. [32] who did not find any improvement in a 2000-m time trial after a 4-week overload training block immediately followed by a 2-week step taper in rowers. The authors estimated the training load from training volume and did not control the training intensity of the sessions and its distribution during the “overload” period. Altogether, these results reinforce the argument that pre-taper training needs to be carefully controlled to obtain the benefits of a taper. This is particularly important in team sports since the usual decrease in training intensity during the two weeks before the championship can be masked by an increased duration of technical/tactical sessions. Training content and load periodization throughout intensive training seem to be key determinants, along with recovery, of performance supercompensation [33].

### Intensive training and taper effects on sleep quality

Players with the lowest sleep quality and the highest sleepiness after intensive training were those diagnosed as F-OR [34, 35]. Even though a previous study questioned whether impaired sleep was a cause or a consequence of an overload period [36], the present results suggest that altered sleep elicited the onset of F-OR, since poor sleep quality (i.e. PSQI > 5 and ESS > 10) was reported at t0, but improved at the end of intensive training (t1) and remained stable during the taper (t2). Thornton et al. [37] assessed the effect of daily training load variations on subsequent sleep quantity in rugby league players, and reported that players expressed a better sleep quantity and quality when acceleration/deceleration loads (measured by GPS) were higher. Nedelec et al. [8] hypothesized that acceleration/deceleration efforts during training may exacerbate players’ perception of fatigue, inducing an increase in sleep quantity and quality, which also appears to be the present case in athletes reporting a disturbed sleep. Furthermore, it could be speculated that poor sleep quality reported in F-OR was a possible cause for the lower supercompensation observed compared to AF, according to the physiological and psychological role of an efficient sleep on post exercise recovery [15].

### Limits

For a proper interpretation of the study, some limitations should be acknowledged. First, elite players’ schedule constraints limited participant availability and led to a small sample size which did not allow us to reach sufficient statistical power. Second, due to the high physical demands of the RHIE test, it was decided to avoid such a test at t0, whereas it was needed to assess the supercompensation effect. Finally, the sanitary rules associated with the Covid-19 pandemic provoke a modification of the training content, which certainly affect the efficacity of the training program.

### Practical applications

The absence of clear RHIE performance benefits of a short-duration taper combined with proactive recovery highlights the importance for coaches and sport scientists to rigorously plan, quantify and control training loads, training content distribution and subsequent player fatigue levels during intensive training periods. Furthermore, the implementation self-reported sleep quality screening during periods of intensified training appears to be an easy way to identify players who are at greater risk to develop a state of functional over-reaching, and also to assess the effects of the taper intervention.

## CONCLUSIONS

In the absence of a strict control of the training load periodization (i.e. training content distribution and training intensity) during intensive training due to the Covid-19 sanitary situation, the level of accumulated fatigue before the taper was unsuitable to obtain clear benefits from this intervention. Nevertheless, this study provided promising results regarding the interaction between training load variation, pre-taper fatigue level and sleep, since poor sleep quality before intensive training appear to predispose the players to the development of functional overreaching. Future studies are required to confirm these results in the context of a well-controlled preseason intensive training period.

## Conflict of Interest

The authors declare no conflict of interest.
